# Panoramic radiographs and quantitative ultrasound of the radius and phalanx III to assess bone mineral status in postmenopausal women

**DOI:** 10.1186/s12903-018-0593-4

**Published:** 2018-07-28

**Authors:** Katarzyna Grocholewicz, Joanna Janiszewska-Olszowska, Magda Aniko-Włodarczyk, Olga Preuss, Grzegorz Trybek, Ewa Sobolewska, Mariusz Lipski

**Affiliations:** 1 Department of Interdisciplinary Dentistry, Pomeranian Medical University in Szczecin, Al. Powstancow Wlkp. 72, 70-111 Szczecin, Poland; 2 Department of Oral Surgery, Pomeranian Medical University in Szczecin, Szczecin, Poland; 3 Department of Dental Prosthetics, Pomeranian Medical University in Szczecin, Szczecin, Poland; 4 Department of Preclinical Conservative Dentistry and Preclinical Endodontics, Pomeranian Medical University in Szczecin, Szczecin, Poland

**Keywords:** Osteoporosis, Quantitative ultrasound, Panoramic radiograph, Radiomorphometric indices

## Abstract

**Background:**

Various mandibular indices have been developed to detect osteoporosis on panoramic radiographs. Quantitative ultrasound (QUS) is a low-cost, radiation-free method to assess bone status. The aim of this study was to compare mandibular morphometric analysis and QUS at the radius and proximal phalanx III finger.

**Methods:**

The study involved 97 postmenopausal women, aged 48.5–71.5y (mean: 55.4). Mandibular morphometric analysis comprised: distance between upper and lower mandibular borders just behind the mental foramen (H), distance: mental foramen - inferior mandibular cortex (IM) and mandibular cortical width at the mental region (MCW). Then, ratios were calculated: MCW/IM = PMI (panoramic mandibular index), H/IM = MR (mandibular ratio). Mandibular cortical index (MCI) was used to classify the morphology of the mandibular cortex.

Bone mineral status assessed using QUS at the radius and proximal phalanx III finger was compared to population mean apical bone mass (T-score).

Linear regression analysis was used for correlations between continuous variables, Pearson’s correlation coefficient r - for variables of normal distribution. Student’s t-test was used to compare variables of normal distribution and for the latter - Mann-Whitney U-test. The level of significance was *p* < 0.05.

**Results:**

Mandibular height was 13.42–34.42 mm. The mean mandibular cortical width was 3.31 mm. Mean values of PMI and MR were 0.33 and 2.57, respectively. Higher mean value of Ad-SoS was found in the radius than in the III finger. Phalanx T-score values ​​were lower than those of the radius. T-score of the radius was < − 1.0 in 22 patients, indicating osteopenia. Basing on phalanx T-score, osteopenia was found in 39 patients. Category C1 of Mandibular Cortical Index was found in 48 women, C2 - in 37 women and C3 - in 12 women. Higher scores of Mandibular Cortical Index were recorded in older women. MCI significantly correlated with the skeletal status (*p* = 0.01) as well as with H, MCW and MR. Phalanx T-score was not correlated to PMI, MR or MCW.

**Conclusions:**

1. Mandibular Cortical Index can be used as a screening tool for detecting osteoporosis.

2. Quantitative ultrasound at the phalanx III constitutes a reliable way of assessing bone status.

**Electronic supplementary material:**

The online version of this article (10.1186/s12903-018-0593-4) contains supplementary material, which is available to authorized users.

## Background

In 1993, osteoporosis was defined as a “disease characterized by low bone mass and microarchitectural deterioration of bone tissue, leading to enhanced bone fragility and a consequent increase in fracture risk”. More recently, the National Institutes of Health (NIH) Consensus Development Panel on Osteoporosis defines osteoporosis as a skeletal disorder characterized by compromised bone strength predisposing a person to an increased risk of fracture. The gold standard in assessing bone mineral density (BMD) is dual-energy X-ray absorptiometry (DXA) [1]. The most recent and non-radiation exposure method of assessing bone mineral status is quantitative ultrasound (QUS). Quantitative ultrasound is a non-invasive and inexpensive method for estimating bone mineral status through measurements performed at skeletal sites with predominance of cortical bone, such as calcaneus (the most validated method) and most recently - proximal phalanges [2–5]. However, no studies reporting on correlations between findings on panoramic radiographs and QUS of the phalanx or radius have been found.

The oral implications of osteoporosis include loss of teeth, loss in alveolar bone height, erosion of inferior mandibular cortex, reduced mandibular inferior cortical width. The earliest suggestion of an association between osteoporosis and oral bone loss was made in 1960 [6–8].

Dental panoramic radiograph is one of the most popular radiographs, performed as a diagnostics image before dental treatment. An important advantage of panoramic radiographs is very low cost in comparison to the expensive DXA technique. Identifying women with low bone mineral density by using panoramic radiographs is a particular topic that has drawn the attention of researchers over the last decade [9]. Osteopenia can be identified by thinning of the cortex at the lower border of the mandible. A number of mandibular cortical indices, including the mandibular cortical index (MCI), panoramic mandibular index (PMI), mental index (MI), antegonial index (AI) and gonial index (GI) have been developed to assess and quantify the quality of mandibular bone mass and to observe signs of resorption on panoramic radiographs for identification of osteopenia [10–13].

The earlier the diagnosis of osteopenia or osteoporosis is made the better is for prophylaxis of bone fractures. The dentists may play an important role in early detection of increased bone fracture risk. Dental radiographs, especially panoramic images, have been used to predict low bone mineral density in patients. It is likely that the clinician may estimate the future risk of fractures and osteoporosis by dental panoramic radiographs [14–20].

The aim of this study was to evaluate the diagnostic efficacy of panoramic radiography using morphometric analysis in early detection of osteopenia and osteoporosis in post-menopausal women and to correlate it with the bone mineral status assessed by QUS at the radius and proximal phalanx III finger.

## Methods

### Study sample

The study involved 97 postmenopausal women, aged 48.5 to 71.5 years (mean age 55.4 years), who were patients of the Department of Integrated Dentistry of Pomeranian Medical University in Szczecin, Poland and had panoramic radiographs made using a Digital Panoramic System (Soredex Cranex 3D, Soredex, Finnland) and a computer (Windows - XP operating system, Service Pack – 3, 64 bit, flat screen LCD display). The panoramic radiographs were made in order to plan the dental treatment. Head position was standardized as much as possible.

The exclusion criteria were: previous diagnosis of osteoporosis, interview history of hysterectomy or oophorectomy, history of medication affecting bone metabolism such as glucocorticoids, anticonvulsants, excessive thyroxin doses, diseases altering bone metabolism such as hyperparathyroidism, multiple myeloma and estrogen replacement therapy.

### Radiographic measurements

**Fig. 1:**
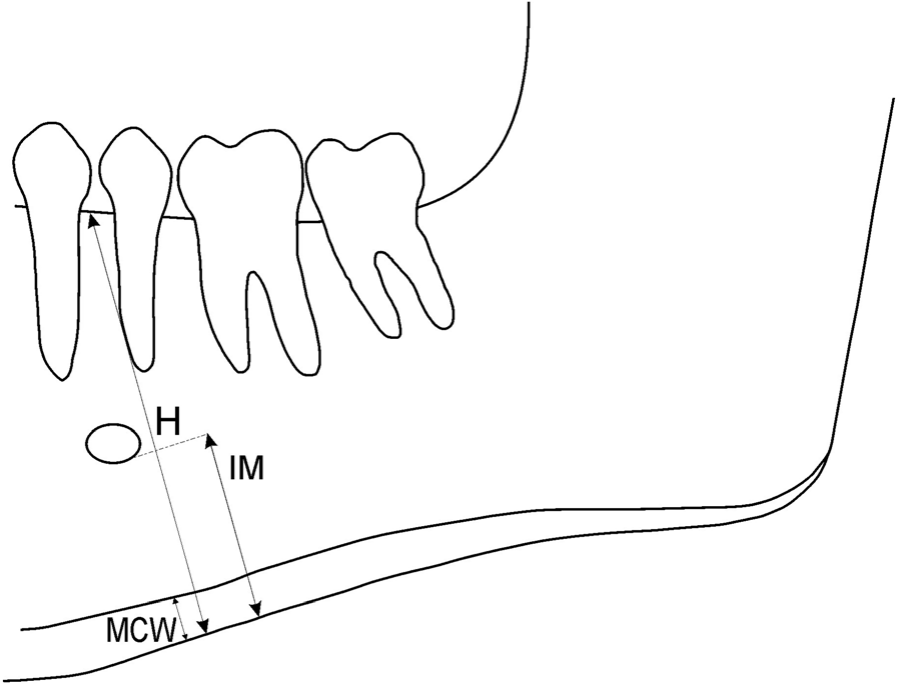
Measurements performed on panoramic radiographs

All the panoramic radiographs were analyzed on the screen of a monitor (2.3-Megapixel Medical Clinical Display, 1920 × 1200 native resolution, DICOM conformance) with subdued lighting condition. One researcher evaluated each image according to patient positioning, head alignment, film density as well as contrast and radiographs with distortion were excluded. All the radiographs were re-analyzed by the same observer after 2 weeks interval. On each radiograph, the following radiomorphometric measurements were made (Fig. 1):

1. Mandibular height (H): the distance between the lower and upper border of mandible, measured just behind the mental foramen
2. IM: distance between the mental foramen and the inferior mandibular cortex
3. Mandibular cortical width at the mental region (MCW): mandibular cortical thickness measured on the line perpendicular to the bottom of the mandible at the middle of the mental foramen.

Based on these measurements panoramic mandibular index (PMI) and mandibular ratio (MR) were calculated. Panoramic mandibular index PMI, was calculated according to Benson et al. [18] as the ratio of MCW/IM,. MR, an indicator of alveolar crest resorption degree developed by Wical and Swoope [19], was calculated as the ratio H/IM.

**Fig. 2:**
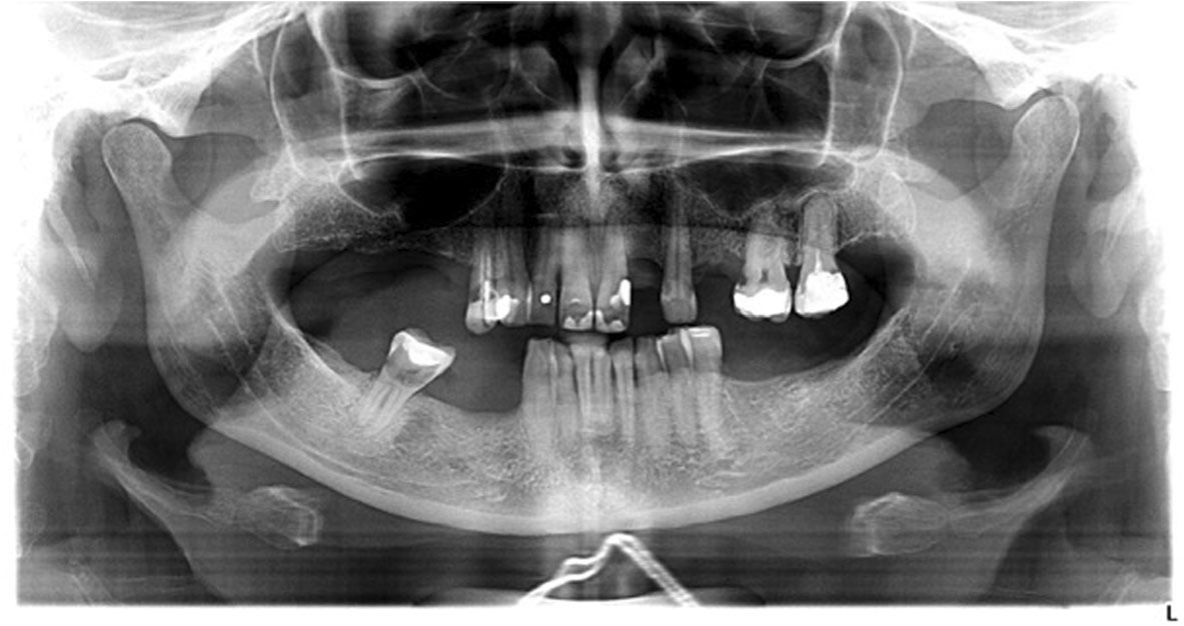
Panoramic radiograph with C1 category of MCI

**Fig. 3:**
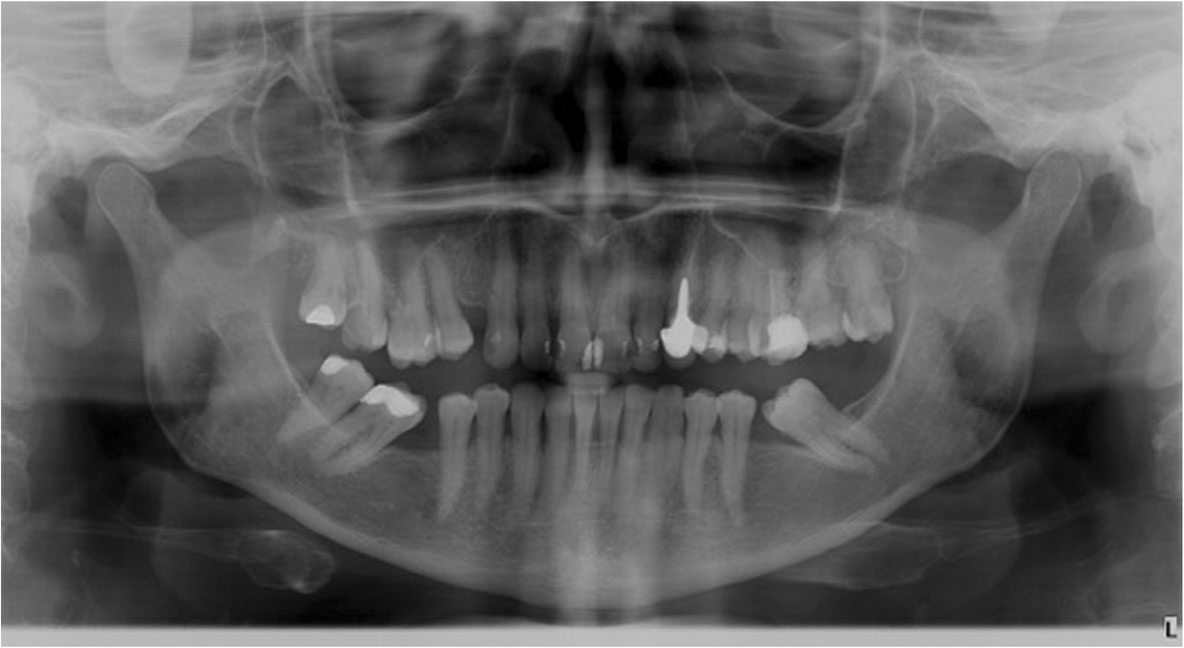
Panoramic radiograph with C2 category of MCI

**Fig. 4:**
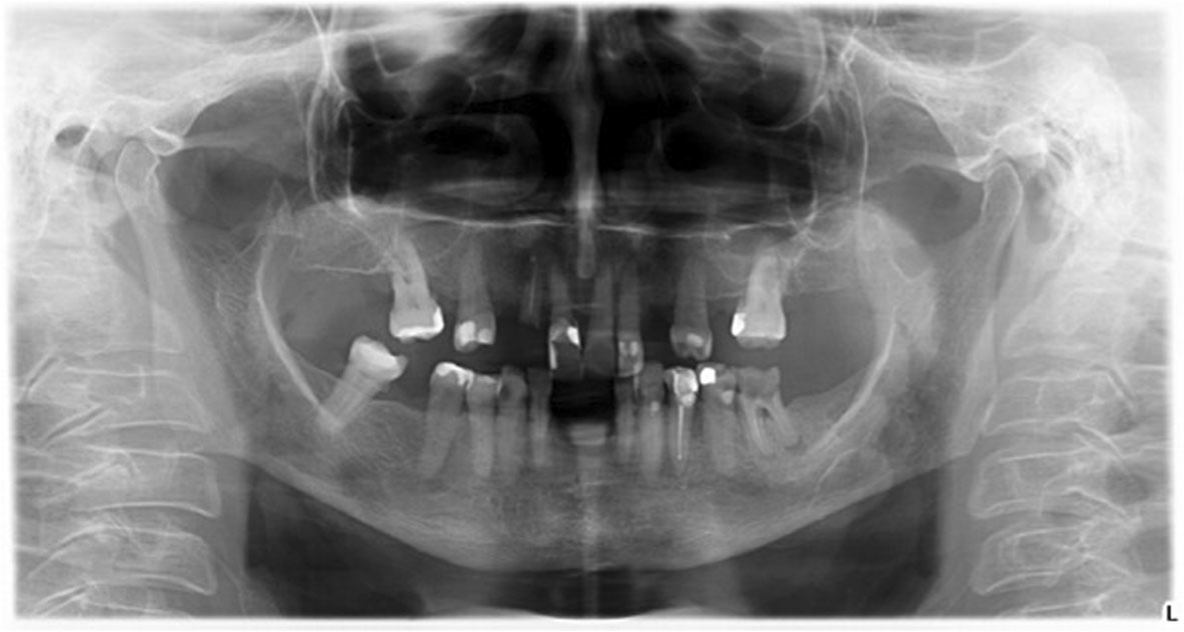
Panoramic radiograph with C3 category of MCI

Moreover, a morphological classification of the mandibular cortical bone has been proceeded on panoramic radiographs. Mandibular cortical index (MCI) according to Klemetti et al. [20] was used to assess morphological changes in the inferior cortex of the mandible. Mandibular cortical shapes were analysed by observing the mandible distally from the mental foramina bilaterally and by categorizing them into one of the following three groups as previously described by Klemetti et al. [20]: “C1 – the endosteal margin of the cortex is even and sharp on both sides (Fig. 2); C2 - the endosteal margin shows semilunar defects (lacunar resorption) or endosteal cortical residues on one or both sides, mild to moderate cortex erosion (Fig. 3); C3 – the cortical layer forms heavy endosteal cortical residues and clearly porous, severely eroded cortex” (Fig. 4).

### Ultrasound bone measurements

In all women, bone mineral density was assessed using an ultrasound bone sonometer Omnisens 7000S (Sunlight Medical Inc.). Measurements were made in the third distal part of radius and distal metaphysis of the proximal phalanx III finger of the non-dominant hand. The sonometer measures the Amplitude-dependent Speed of Sound - Ad-SoS, expressed in m/s. The result of QUS was related to the mean values in population and showed as a number of standard deviations from mean apical bone mass of young adults of the same sex (T-score). In the assessment of the skeletal system the most useful is to compare the results of the study with a mean apical bone mass of the population (T-score). According to World Health Organization, the following criteria were applied to assess osteoporosis: T-score ± 1.0 was considered normal; − 2.5 ≤ T-score < − 1.0 was a sign of osteopenia; T-score < − 2.5 indicated osteoporosis.

### Statistical analysis

The results were analyzed using the statistical package STATISTICA 6.0. Linear regression analysis was used to check for correlations between continuous variables. Pearson’s correlation coefficient r was calculated between variables of normal distribution. Student’s t-test was used to analyse statistical significance of differences between variables of normal distribution and for the latter - Mann-Whitney U-test was used. The level of significance has been established at *p* < 0.05.

## Results

The results of measurements and radiomorphometric indices of the mandible are summarized in Table 1. Raw data is provided as an additional Excel file. Mandibular height ranged between 13.42 and 34.42 mm. The mean mandibular cortical width was 3.31 mm. Mean values of PMI and MR were 0.33 and 2.57, respectively.

**Table 1:** Distribution of radiomorphometric measurements and mandibular indices

Parameter	*n*	Mean	Median	Minimum	Maximum	SD
H (mm)	97	25.22	25.58	13.42	34.42	3.20
IM (mm)	97	9.96	9.85	6.42	15.23	1.61
MCW (mm)	97	3.31	3.38	1.69	5.38	0.67
PMI	97	0.33	0.33	0.05	0.52	0.07
MR	97	2.57	2.54	1.51	3.78	0.40

The parameters describing the skeletal status are summarized in Table 2. Higher mean value of Ad-SoS was observed in the distal part of radius than in the proximal phalanx III finger.

**Table 2:** The skeletal status in examined women

Parameter	*N*	Mean	Median	Minimum	Maximum	SD
Phalanx Ad-SoS (m/s)	97	3941	3976	3337	4384	194
Phalanx T-score	97	−0.65	− 0.4	− 3.60	2.30	1.26
Radius Ad-SoS (m/s)	97	4152	4160	3853	4601	130
Radius T-score	97	−0.18	−0.2	−3.2	4.4	1.33

T-score values were lower for the phalanx than for the radius.

Ultrasonographic measurements showed that the T-score of the radius was < − 1.0 in 22 patients, indicating greater than a physiological loss of bone mass, meaning osteopenia. However, basing on the phalanx T-score, osteopenia was found in 39 patients. These results are presented in Table 3.

**Table 3:** Distribution of the study group according to T-score values

Parameter	T-score ≥ − 1 *n*	T-score < − 1 *n*	Total *n*
Phalanx	58	39	97
Radius	75	22	97

Category C1 of Mandibular Cortical Index was found in 48 women, category C2 in 37 women and category C3 in 12 women. Higher categories of Mandibular Cortical Index were recorded in older women (Table 4).

**Table 4:** MCI categories according to the age

MCI	*n*	Age (years)				SD
		Mean	Median	Minimum	Maximum	
C1	48	54.8	54	48.5	67	4.3
C2	37	55.3	54	50	69	4.7
C3	12	58.5	55.5	50	71.5	7.9
Total	97	55.4	54	48.5	71.5	5.0

Table 5. shows the distribution of patients in each MCI group, according to the phalanx and radius T-score. Phalanx T-score revealed more women with low bone mass and, therefore, it was used for the determination of changes in bone.

**Table 5:** Distribution of subjects in individual Mandibular Cortical Index groups according to the phalanx and radius T-score

MCI	Phalanx T-score ≥ − 1 *n*	Radius T-score ≥ − 1 *n*	Phalanx T-score < − 1 *n*	Radius T-score < − 1 *n*
C1	32	41	16	7
C2	24	28	13	9
C3	2	6	10	6

The analysis of results obtained revealed 39 patients with osteopenia according to the phalanx T-score. The highest porosity inferior cortex (C3) was observed in 12 patients and in 10 of them phalanx T-score was < − 1. The mild eroded cortex (C2) was observed in 37 women and 13 of them showed osteopenia. Correlations between MCI and bones parameters indicated that in patients with a reduction of bone mass, the mandibular cortex shape was characterized by a higher porosity.

Mandibular Cortical Index significantly correlated with the skeletal status and mandibular height, mandibular cortical width as well as mandibular ratio. These relationships are presented in Table 6.

**Table 6:**
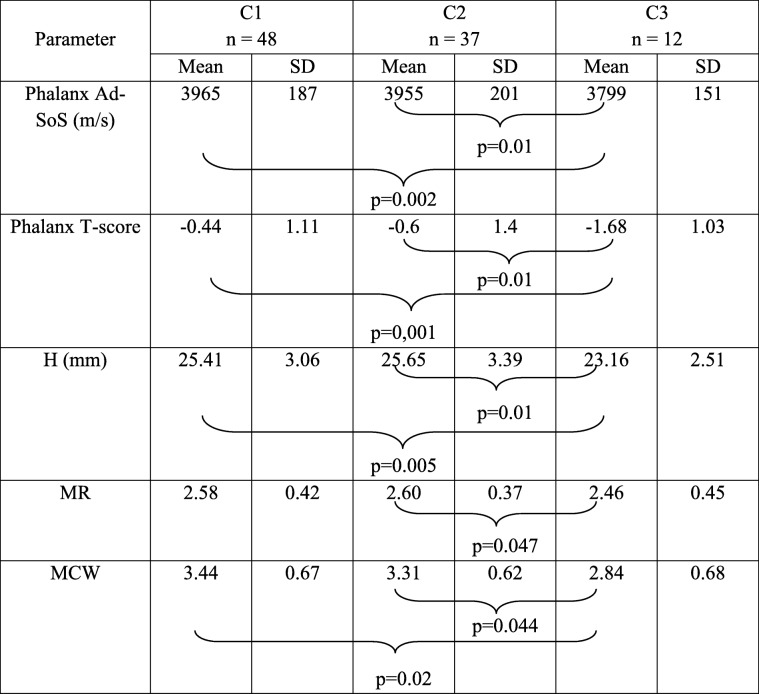
Mean values of observed parameters according to Mandibular Cortical Index

The Ad-SoS and T-score in phalanx, mandibular height, Mandibular Ratio and Mandibular Cortical Width were significantly lower in patients with higher category of MCI.

In women with C3 category of mandibular cortical index, phalanx Ad-SoS, phalanx T-score and the mandibular cortical width were significantly lower than in women with C2 and C1 category.

The present study showed correlations between mandibular cortical shape (MCI) and the status of bones as well as MR and MCW. Phalanx T-score was not correlated with other indexes relating radiomorphometric measurements PMI, MR and MCW (Table 7).

**Table 7:** Pearson’s correlation coefficients

Characteristics	Pearson’s correlation coefficient r	*p* value
MCI versus phalanx Ad-SoS	−0.22	< 0.05
MCI versus phalanx T-score	−0.27	< 0.05
MCI versus MR	0.25	< 0.05
MCI versus MCW	−0.26	< 0.05

## Discussion

Panoramic radiography is frequently performed before dental treatment, especially in older patients, to assess not only dental status but also the status of bones. Many studies suggested that incidental findings detected on these radiographs might be helpful to identify patients with low bone mineral density [13–17, 21]. This is the first study to compare measurements from panoramic radiographs and the radiation-free method of QUS of the phalanx and radius.

No correlations between MCW and phalanx as well as between Ad-SoS and phalanx T-score were found in the present study. Moreover, both PMI and MR proved to be ineffective in the screening of osteopenia/osteoporosis in women. These results are consistent with those by other authors, who emphasized poor utility of PMI and MR, but usefulness of MCI and MCW as screening tools for osteoporosis. Bhatnagar et al. [22] found a weak correlation between PMI and BMD. The same study showed that the degree of mandibular cortical shape erosion significantly correlated with BMD and concluded that the combined mandibular cortical findings (mandibular cortical shape erosion and mandibular cortical width) on panoramic radiographs were effective indicators of osseous changes in postmenopausal osteoporosis.

A similar evaluation was performed by Benson et al*.* [23], who used PMI to compensate for the vertical magnification that differs among various panoramic machines, but found a very weak correlation between the index and BMD in spite of the fact that PMI is inclusive of other variable i.e., half mandibular width. Therefore Benson et al*.* [23] used MCW, instead of PMI as an effective indicator. Klemetti et al. [23] also found that linear correlation of the panoramic mandibular index with all bone mineral density values was weak. Patients with reduced bone mass showed lower height of mandible and thereupon also MR.

Stagraczynski et al. [24] showed that PMI and MR are not adequate radiological markers of vertebral bone loss in postmenopausal women. However, measurements of the distance between the inferior margin of the mental foramen and the inferior mandibular cortex did correlate with the degree of lumbar BMD deficiency.

Lee et al. [25] concluded that simple visual estimation of the mandibular inferior cortex width on panoramic radiographs might be useful for identifying postmenopausal women with low BMD. In similar studies Ohtsuki et al., [26] as well as Horner and Devlin [27] also found that mandibular cortical width significantly correlated with BMD,.

The lower specificity of cortical width in identifying low BMD, comparing to cortical shape was confirmed in the study by Khojastehpour et al. [28]. They demonstrated significant associations between BMD and MCW and MCI and concluded that postmenopausal women with thin or eroded mandibular inferior cortex may have an increased risk of low BMD or osteoporosis. Bollen et al. [29] observed that subjects with a self-reported history of osteoporotic fractures tend to have increased resorption and thinning of the mandibular lower cortex. In a study by Gulsahi et al. [30], it was stated that patients with C3 type of MCI should be considered as high-risk individuals for osteoporosis irrespective of age and gender. The usefulness of a visual estimation of the mandibular cortical bone integrity from panoramic radiographs for identifying postmenopausal women at high risk for osteoporosis has been confirmed by Geary et al. [31] as well as Alapati et al. [32].

Some authors emphasize that more lengthy training and experience in using the MCI would be needed for it to be effective as a diagnostic tool in general dental practice [33, 34].

In most studies, the diagnosis of osteoporosis is based on bone mineral density measured by dual-energy X-ray absorptiometry (DXA), but this technique is not a practical and economical one [22–25, 27, 28]. Quantitative ultrasound at the hand phalanges has been less popular [2–5], so far, but the present study indicates, it constitutes a reliable method of assessing bone status.

Recently, studies have been published describing the use of radiomorphometric indicators with CBCT and computerized estimation of the risk of osteoporosis [35–37]. This could be a promising direction in the development of dental diagnostics for identifying women with low bone mineral density. However, CBCT to evaluate the bone density of jaws, despite the lower radiation dose and cost, is not useful when absolute values are taken into consideration; a conversion ratio needs to be applied [38].

## Conclusions

The present study showed a correlation between mandibular cortical shape and bone status. MCI can be effectively assessed on panoramic radiographs, hence could be used as a screening tool for determining osteoporosis. This index, very simple for evaluation, may possibly be used as a potential screening tool in identifying individuals with osteoporosis. As routinely requested in dental offices, dental panoramic radiography has an important role in referring patients for osteoporosis investigation.

The simple method of quantitative ultrasound at the hand phalanges constitutes a reliable way of assessing bone status.

## Additional file

Additional file 1: Osteoporosis. Raw data. Individual values of all parameters analyzed are provided in Excel. (XLSX 34 kb)

## Abbreviations

Ad-SoS: Amplitude-dependent Speed of Sound
AI: Antegonial index
BMD: Bone mineral density
DXA: Dual-energy X-ray absorptiometry
GI: Gonial index
H: Mandibular height just behind the mental foramen
IM: Distance between the mental foramen and the inferior mandibular cortex
MCI: Mandibular cortical index
MCW: Mandibular cortical width at the mental region
MI: Mental index
MR: Mandibular ratio (H/IM)
NIH: National Institutes of Health
PMI: Panoramic mandibular index
PMI: Panoramic mandibular index (MCW/IM)
QUS: Quantitative ultrasound
